# Extensive bullous lichen sclerosus et atrophicus*

**DOI:** 10.1590/abd1806-4841.20164398

**Published:** 2016

**Authors:** Jelica Vukicevic

**Affiliations:** 1 Clinical Center of Serbia, Faculty of Medicine, University of Belgrade – Belgrade, Serbia

**Keywords:** Blister, Chloroquine, Doxycycline, Lichen sclerosus et atrophicus, Hydroxychloroquine

## Abstract

Lichen sclerosus et atrophicus is a chronic disease of unknown etiology characterized by atrophic and sclerotic plaques in both genital and extragenital regions. Extensive bullous lichen sclerosus et atrophicus (BLSA) is a severe variant of the disease with no widely accepted treatment. We present a 63-year-old woman with extensive extragenital, ivory-colored, atrophic plaques on her trunk and extremities and disseminated hemorrhagic bullae. The patient was unsuccessfully treated with standard topical corticosteroid therapy, doxycycline and chloroquine. According to the literature, there is little evidence of the efficacy of doxycycline and hydroxychloroquine in the treatment of BLSA. We report a rare case of extensive BLSA that is unresponsive to these drugs.

## INTRODUCTION

Lichen sclerosus et atrophicus (LSA) is a chronic mucocutaneous disease of unknown etiology first described by Hallopeau in 1887. It is commonly presented as white, atrophic, porcelain-like plaques in the anogenital region in postmenopausal women. Extragenital lesions of similar morphology may occasionally be present. Bullous lichen sclerosus et atrophicus (BLSA) is a rarely reported disease whose clinical appearance and pathological findings are rather characteristic. Bullous lesions are usually transient and heal before the appearance of typical plaques, which are more resistant to treatment.^1^ Extensive BLSA is the most severe form of the disease with no widely accepted treatment, although various therapeutic options have been suggested. We present a rare, extensive, extragenital BLSA that is unresponsive to doxycycline and chloroquine.

## CASE REPORT

**Figure 1:**
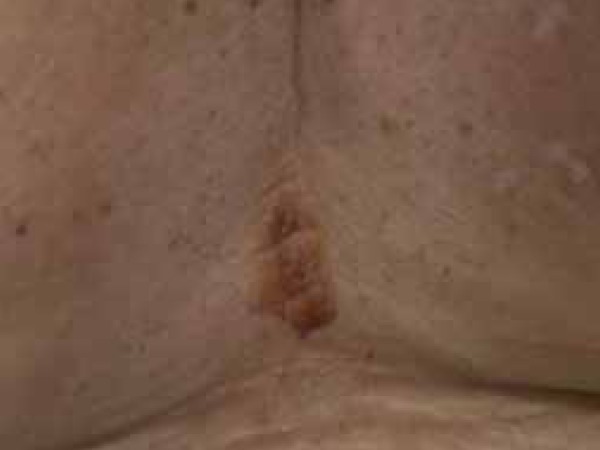
Hemorrhagic bulla within pre-existing atrophic lesion involving her low back

**Figure 2:**
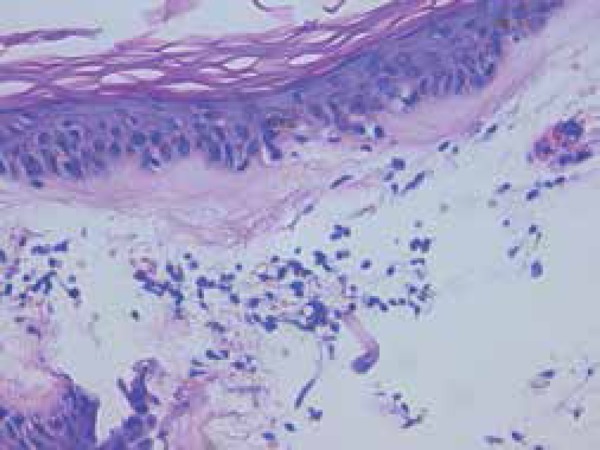
Histopathology showing epidermal atrophy, focal basal liquefaction, edema with dermoepidermal clefting and homogenization of collagen in the papillary dermis (Hematoxylin - eosin, x400)

**Figure 3:**
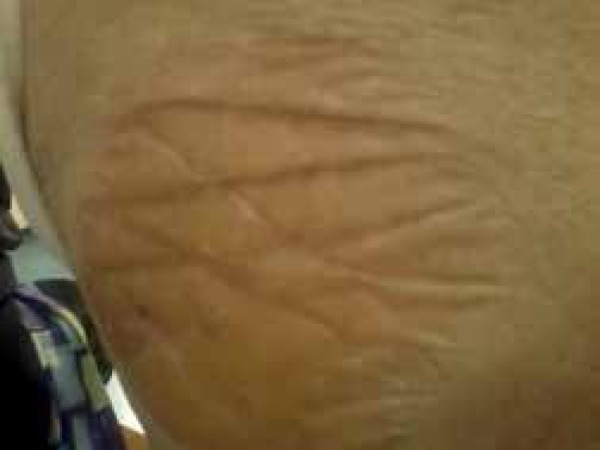
Bulla in the gluteal region

**Figure 4:**
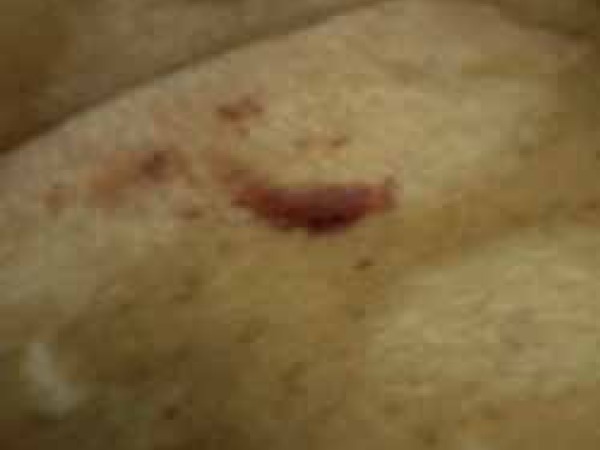
New hemorrhagic bulla on the abdomen

**Figure 5:**
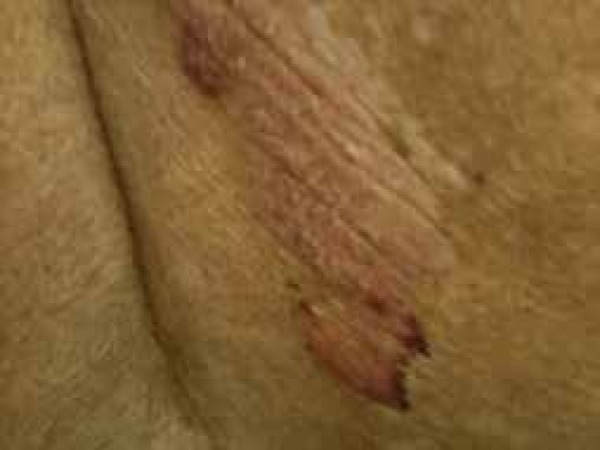
New haemorrhagic bulla on the back

We report a 63-year-old woman with a few months' history of cutaneous lesions on her trunk and extremities. Histopathological analysis of the lesions revealed lichen sclerosus et atrophicus (LSA), which were treated with topical corticosteroids. Personal and family history was unremarkable. Our first examination revealed multiple, disseminated, hypopigmented and hyperpigmented atrophic and sclerotic plaques on her trunk and extremities. The patient presented a 6x8cm fragile, hemorrhagic bulla within an atrophic plaque on her lower back (Figure 1). We performed an incisional biopsy of the bulla that revealed epidermal atrophy, a subepidermal blister with marked edema of the papillary dermis and homogenization of collagen (Figure 2). Blood count, biochemical parameters and coagulation studies were within the normal range. Immunological analyses revealed positive (1:160) antinuclear antibodies (ANAs) by indirect immunofluorescence (Hep-2-cells) (homogenous pattern), and negative anti-ssDNA, anti-dsNA, anti SSA/Ro,Scl-70 and anti-SSB/ La. IgG and IgM (EIA) antibodies were negative for Lyme's disease. Chest X-ray, abdomen ultrasound and gynecological examination showed no pathological findings. The patient was initially treated with doxycycline 100 mg/ day and locally applied 0, 5% clobetasol. The bulla resolved within three weeks, but new hemorrhagic bulla appeared in the same place together with painful, non-hemorrhagic bullae on both gluteal regions (Figure 3). Chloroquine was administered in daily doses of 250 mg. After a month, we observed some improvement of the skin lesion and resolution of the bulla on the back, but soon after, new multiple hemorrhagic bullae appeared on the trunk (Figures 4 and 5). We observed no erosion healing on the sites of previous bullae in the gluteal regions, where shallow, painful ulcers were formed. The patient refused the advised hospitalization and further follow-up appointments.

## DISCUSSION

The bullous variant of lichen sclerosus et atrophicus (LSA) is a rare disorder thought to occur due to extensive vacuolar degeneration of the basal epidermal layer resulting in fragility of the dermal-epidermal union and marked edema in the papillary dermis. It is often accompanied by disruption and loss of collagen support of the dermal capillaries and consequent hemorrhage within the bullae.^1^ Bullous LSA is most common in adult women but also occurs in men and children. Although the etiology of bullous LSA remains obscure, many factors have been suggested. Low sex hormone output, loss of androgen receptor expression, random inactivation of the androgen receptor gene, autoimmunity with type II diabetes mellitus and *Borrelia* infection are considered possible causes.^2^ Kimura A. suggests that minor trauma may be the cause of blister formation in extragenital LSA.^3^ Breier F. reported that *Borrelia afzelii* was isolated and identified by polimerase chain reaction (PCR) from a skin lesion in a seronegative patient with generalized BLSA.^4^ We observed no underlying spirochetal infection, autoimmune disease, diabetes, or history of trauma in our patient. The appearance of the non-hemorrhagic bullae in both gluteal regions in our patient may be a result of Koebner phenomenon. Chronic pressure had previously been reported to act as a trigger for the Kobner response in lichen sclerosus.^5^

According to the literature, in a few cases of both genital and extragenital BLSA, bullae suppression was achieved by conventional treatment with potent topical corticosteroids.^1,2^ Although numerous therapeutic options have been suggested for progressive BLSA, there is no definitive treatment for the disease. Rarity of the disease makes it difficult to evaluate proposed therapies. Only two reports of treatment of BLSA with hydroxycloroquine are available, only one recording improvements of BLSA with the drug.^6,7^ Madan and Cox gave evidence of gradual clearance of the skin lesions and resolution of the bullae using doxycycline in extensive bullous lichen sclerosus, while other authors successfully used corticotropin.^8,9^ Although etretinate has been used for genital disease, the drug proved unhelpful and even worsens generalized forms.^7^ Impressive success in the treatment of extensive BLSA was achieved with ceftriaxone and systemic corticosteroids in a *Borrelia afzelii* positive patient. ^4^ Treatment with doxycycline and chloroquine, in the presented case, was based on the sporadic bullae occurrence in the beginning and previous reports of successful use of this drugs in extensive BLSA. However, due to the disease's progression, treatment with pulsed high-dose corticosteroids combined with low-dose methotrexate was considered. ^10^

This is another rare case of extensive BLSA that has been resistant to conventional treatment with topical potent corticosteroids, doxycycline and chloroquine.
